# Clinical presentations and outcomes of pancreaticobiliary maljunction in different pediatric age groups

**DOI:** 10.1186/s12887-023-04248-y

**Published:** 2023-08-26

**Authors:** Hui-min Mao, Shun-gen Huang, Yang Yang, Tian-na Cai, Lin Fang, Wan-liang Guo

**Affiliations:** 1 Department of Radiology, Children’s Hospital of Soochow University, No. 92 Zhongnan Street, Suzhou, China; 2 Pediatric Surgery, Children’s Hospital of Soochow University, Suzhou, China

**Keywords:** Age, Children, Clinical presentation, Outcomes, Pancreaticobiliary maljunction

## Abstract

**Background:**

Pancreaticobiliary maljunction (PBM) is a congenital defect, with risk of developing various pancreaticobiliary and hepatic complications. The presentations of PBM in children and adults are believed to be different, but studies on PBM children of different age groups are limited. This study was to evaluate clinicopathologic characteristics and outcomes in PBM children of different ages.

**Methods:**

A total of 166 pediatric patients with PBM were reviewed retrospectively. Clinicopathological, imaging, laboratory, surgical, and follow-up data were collected and analyzed. The patients were divided into three age groups, namely, group A (< 1 year, n = 31), group B (1–3 years, n = 63), and group C (> 3 years, n = 72).

**Results:**

The major clinical manifestation was jaundice in group A and abdominal pain and vomiting in groups B and C. Acute pancreatitis was more often seen in group C than group A. The length of common channel was significantly longer in group C than group A, while the maximum diameter of common bile duct in group C was smaller than that in group A. Cholangitis and cholecystitis were more commonly performed in groups B and C, while hepatic fibrosis in group A. Whether preoperatively or postoperatively, group C was more likely to have elevated serum amylase, while groups A and B were more likely to present with abnormal liver function indicators, including the increase of aspartate transaminase, alanine transaminase, and gamma-glutamyl transpeptidase.

**Conclusion:**

Presentation of PBM varies among different pediatric age groups, thus suggesting that targeted management should be carried out according to these differences.

## Background

Pancreaticobiliary maljunction (PBM) is a congenital defect in which the pancreatic and bile ducts join outside the duodenal wall and they usually form a long common channel [1]. PBM is more common in Asian countries than in western countries, and most PBMs develop in children [2, 3]. Due to this anatomical abnormality, Oddi sphincter fails to regulate the function of the pancreaticobiliary junction, leading to bidirectional reflux of pancreatic juice and bile [4]. Bile flowing back to the pancreatic duct can cause pancreatitis, and reflux of pancreatic juice can damage the bile duct epithelium, resulting in biliary inflammation, stones and even biliary cancer [5]. Therefore, early diagnosis and treatment are crucial for PBM patients.

Typically, PBM with biliary dilatation, known as choledochal cyst (CC), presents in a child as abdominal pain, abdominal mass, and jaundice [6]. However, few patients present with these three typical clinical manifestations, and clinical manifestations among different age groups often differ. Previous studies have focused on the differences in clinical symptoms and surgical outcomes between pediatric and adult patients [7, 8], but a comparison of PBM children of different age groups has been rarely reported [9]. Therefore, the main goal of this study was to evaluate the presentation, pathological and surgical outcomes, and dynamic changes of relevant laboratory indicators preoperatively and postoperatively in PBM children of different age groups. In doing so, the natural history of PBM disease and its long-term effects can be fully understood, ensuring better management of PBM in children of different age groups.

## Methods

### Patient population

**Fig. 1:**
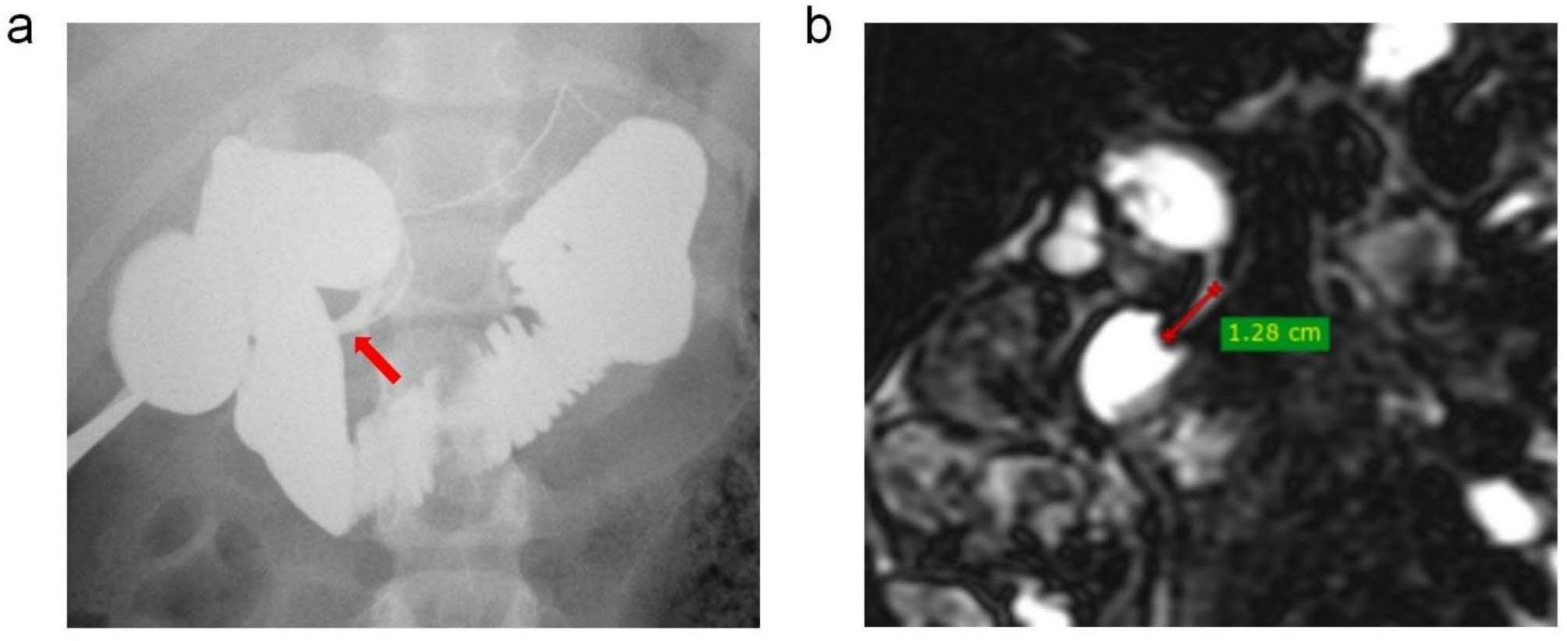
Imaging of pancreaticobiliary maljunction. Intraoperative cholangiography (**a**) and magnetic resonance cholangiopancreatography (**b**) show that the pancreatic and common bile ducts join outside the duodenal wall (arrow), forming a long common channel

This study was approved by the Medical Ethics Committee of the Children’s Hospital of Soochow University, which waived the informed consent requirement because of the retrospective nature of the study and minimal risk to participants. We retrospectively reviewed the medical records of all of the hospitalized children with PBM at the Children’s Hospital of Soochow University between January 1, 2010, and December 31, 2021. PBM was diagnosed based on the recommended diagnostic criteria: an abnormally long common channel between the pancreatic and bile ducts and/or an abnormal union between the pancreatic and bile ducts, as confirmed by surgery or imaging examination (Fig. 1) [10]. Patients without surgical treatment and cases with incomplete clinical, imaging, and laboratory records were excluded. Finally, a total of 166 children were included, and median follow-up was 22.5 months (range, 12–132 months).

In this study, all of the included patients were < 15 years old. According to age at the time of surgery, the patients were divided into the following three groups: group A (< 1 year, n = 31), group B (1–3 years, n = 63), and group C (> 3 years, n = 72).

### Data collection

Preoperative clinical data, including age, sex, symptoms, and related complications, were collected on review of medical records. Imaging data, including ultrasonography, abdominal contrast-enhanced computed tomography (CT), magnetic resonance cholangiopancreatography (MRCP), and intraoperative cholangiography (IOC) were obtained. According to the classical Komi’s method [11], PBM was classified into three distinct types based on MRCP and IOC. In children, based on the maximum diameter of the common bile duct (CBD), PBM was classified as either dilatation type (> 5 mm) or no dilatation type (≤ 5 mm) [12]. Then, PBM with biliary dilatation, known as congenital biliary dilatation or CC, was classified according to Todani’s classification [13]. By observing preoperative MR and CT images, relevant parameters of the bile duct and hepatic vessels were measured. The longest length of the common channel was measured on the coronal plane of MRCP, a straight-line distance from the point of confluence of the bile duct and pancreatic duct to the duodenal wall (Fig. 1). Imaging features and measurements were assessed by two independent experienced radiologists, blinded to clinical and laboratory data.

The baseline, pre-, peri- and post-operative laboratory values, including serum indicators of liver function and serum amylase levels, were recorded. Surgical information and histopathological data were also collected. In this study, all of the included patients underwent excision of the extrahepatic bile duct and Roux-en-Y hepaticojejunostomy. Liver biopsy was simultaneously performed in appropriate cases to evaluate the grade of hepatic fibrosis using the Metavir scoring system [14]. Liver fibrosis was scored from F0 to F4: F0, no fibrosis; F1, portal fibrosis without septa; F2, portal fibrosis with rare septa; F3, numerous septa without cirrhosis; and F4, cirrhosis. Patients were subsequently classified as either having liver fibrosis (F1-F4) or non-fibrosis (F0).

Complications occurring within 30 days postoperatively were considered early complications, and those occurring after 30 days were marked as late complications. Postoperative follow-up results were obtained through clinical records or contact with the patients and their family members over telephone.

### Statistical analysis

All of the statistical analyses were conducted using IBM SPSS 26.0 (Armonk, NY, USA) and GraphPad Prism 8.0 (GraphPad Software, Inc., La Jolla, CA, USA). Counting data were presented as frequency and percentage (%) and analyzed by the Chi-square (χ2) test or Fisher’s exact test. Continuous variables complying with normal distribution were expressed as mean ± standard deviation (SD) and analyzed by one-way analysis of variance. Skewed distributed continuous variables were reported as median and interquartile range (IQR) and analyzed by Kruskal–Wallis test. *P* < 0.05 was considered to be the threshold of significance.

## Results

### Preoperative clinical characteristics

Preoperative clinical and imaging findings of the three different pediatric age groups are presented in Table 1. The median (IQR) age at operation was 4.6 (2.0–9.0) months in group A, 1.9 (1.4–2.5) years in group B, and 5.0 (4.0–7.8) years in group C, with female predominance in all of the three groups. The most common Komi classification of PBM was P-B type (49.4%), followed by B-P type (42.8%). The great majority of patients had PBM with biliary dilatation (94.6%), and all of the children with PBM in group A had biliary dilatation. The distributions of the PBM, CBD, and Todani classifications among the three groups were similar (all *P* > 0.05).

**Table 1:** Preoperative clinical and imaging findings of the three groups

	ALL (n = 166)	Group A (n = 31)	Group B (n = 63)	Group C (n = 72)	Statistical value	*P*-value
Age, median (IQR)	2.8 y (1.3–4.7 y)	4.6 m (2.0–9.0 m)	1.9 y (1.4–2.5 y)	5.0 y (4.0-7.8 y)		
Sex					0.119	0.942
Male, n (%)	35 (21.1)	6 (19.4)	13 (20.6)	16 (22.2)		
Female, n (%)	131 (78.9)	25 (80.6)	50 (79.4)	56 (77.8)		
Type of PBM, n (%)					3.411	0.497
B-P type	71 (42.8)	17 (54.8)	28 (44.4)	26 (36.1)		
P-B type	82 (49.4)	12 (38.7)	31 (49.2)	39 (54.2)		
Complex type	13 (7.8)	2 (6.5)	4 (6.3)	7 (9.7)		
Type of CBD, n (%)					2.275	0.323
With dilatation	157 (94.6)	31 (100.0)	58 (92.1)	68 (94.4)		
No dilatation	9 (5.4)	0 (0.0)	5 (7.9)	4 (5.6)		
Todani classification, n (%)^#^					4.266	0.118
I	76 (48.4)	18 (58.1)	22 (37.9)	36 (52.9)		
IVa	81 (51.6)	13 (41.9)	36 (62.1)	32 (47.1)		
Clinical findings						
Abdominal pain, n (%)	107 (64.5)	4 (12.9)	37 (58.7)	66 (91.7)	60.133	0.000*^abc^
Jaundice, n (%)	27 (16.3)	12 (38.7)	11 (17.5)	4 (5.6)	17.596	0.000*^b^
Fever, n (%)	24 (14.5)	6 (19.4)	11 (17.5)	7 (9.7)	2.542	0.283
Vomiting, n (%)	85 (51.2)	6 (19.4)	36 (57.1)	43 (59.7)	15.566	0.001*^ab^
Biliary perforation, n (%)	5 (3.0)	2 (6.5)	3 (4.8)	0 (0.0)	4.609	0.084
Gallstones, n (%)	62 (37.3)	6 (19.4)	26 (41.3)	30 (41.7)	5.277	0.071
CBD stones, n (%)	44 (26.5)	7 (22.6)	15 (23.8)	22 (30.6)	1.086	0.581
Association with acute pancreatitis, n (%)	18 (10.8)	0 (0.0)	5 (7.9)	13 (18.1)	8.268	0.012*^b^
Pancreatic duct dilatation, n (%)	12 (7.2)	0 (0.0)	5 (7.9)	7 (9.7)	3.133	0.199
CT or MR measurements						
Length of common channel (mm), median (IQR)	9.8 (8.7, 12.0)	9.3 (9.0, 10.0)	9.8 (9.0, 10.0)	11.2 (8.2, 13.9)	10.006	0.007*^b^
Diameter of CBD (mm), median (IQR)	24.1 (16.3, 34.3)	28.8 (22.5, 47.5)	24.4 (15.3, 38.2)	21.1 (15.1, 29.7)	8.716	0.013*^b^
Distance between cystic duct and the confluence of CHD (mm), median (IQR)	17.2 (12.9, 21.7)	14.2 (11.0, 22.1)	18.2 (14.5, 22.3)	17.3 (12.5, 21.6)	4.469	0.107
Diameter of hepatic artery (mm)	2.8 ± 0.6	2.3 ± 0.5	2.9 ± 0.6	3.0 ± 0.5	15.912	0.000*^ab^
Diameter of portal vein (mm)	7.2 ± 1.6	5.7 ± 1.0	6.7 ± 0.9	8.3 ± 1.6	55.050	0.000*^abc^

Abbreviations: IQR, interquartile range; PBM, pancreaticobiliary maljunction; CBD, common bile duct; CT, computed tomography; MR, magnetic resonance; CHD, common hepatic duct

* Significance level is < 0.05; ^a^*P* < 0.05 (group A vs. group B); ^b^*P* < 0.05 (group A vs. group C); ^c^*P* < 0.05 (group B vs. group C)

^#^ Only patients with PBM with biliary dilatation were classified by Todani’s classification

**Fig. 2:**
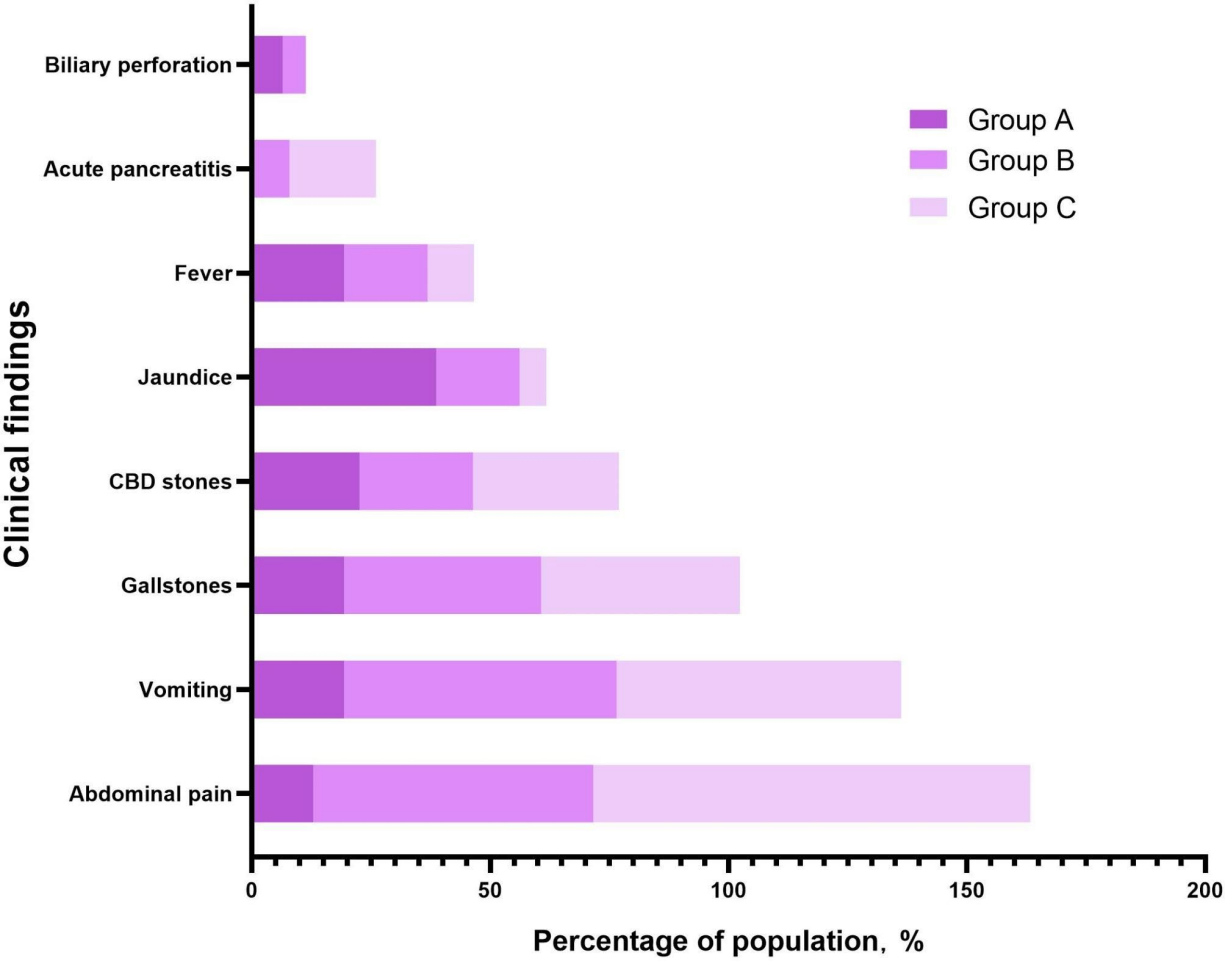
Clinical findings associated with pancreaticobiliary maljunction in the three groups. Jaundice was more common in group A; abdominal pain and vomiting were more common in groups C and B. Abbreviation: CBD, common bile duct

The most common symptom in groups C and B was abdominal pain (91.7% and 58.7%, respectively), followed by vomiting (59.7% and 57.1%, respectively), and the incidence rates were significantly higher than those in group A (12.9% and 19.4%; all *P* < 0.05). The frequency of abdominal pain in group C was also greater than that in group B (91.7% vs. 58.7%; *P* < 0.05). Jaundice was the major clinical presentation in group A, and its incidence in group A was significantly higher than that in group C (38.7% vs. 5.6%; *P* < 0.05). Group C was more likely than group A to have acute pancreatitis (18.1% vs. 0.0%; *P* < 0.05). However, the three groups showed no differences in the proportion of other clinical findings, including fever, biliary perforation, gallstones, and CBD stones (all *P* > 0.05) (see Table 1; Fig. 2).

The length of the common channel was significantly longer in group C than in group A (median, 11.2 mm vs. 9.3 mm; *P* < 0.05; Table 1), while the maximum diameter of the CBD in group C was smaller than that in group A (median, 21.1 mm vs. 28.8 mm; *P* < 0.05; Table 1). As expected, the diameter of hepatic vessels increased with increasing age. The diameter of the hepatic artery and portal vein was significantly smaller in group A than in the other two groups (*P* < 0.05; Table 1).

### Operative therapies and histopathological results

Most patients (n = 146, 88.0%) underwent laparoscopic surgery, and there was no significant difference in surgical approach among the three groups (*P* > 0.05). Meanwhile, operative time and postoperative length of stay were equivalent among the three age groups (*P* > 0.05). However, group C was more likely to have higher intraoperative blood loss than group A (76.6 ± 93.7 mL vs. 42.0 ± 13.1 mL; *P* < 0.05; Table 2).

**Table 2:** Operative and histopathologic results of the three groups

	ALL (n = 166)	Group A (n = 31)	Group B (n = 63)	Group C (n = 72)	Statistical value	*P*-value
Surgical approach					3.559	0.454
Laparoscopy, n (%)	146 (88.0)	27 (87.1)	53 (84.1)	66 (91.7)		
Laparotomy, n (%)	16 (9.6)	3 (9.7)	9 (14.3)	4 (5.6)		
Laparoscopy conversion to laparotomy, n (%)	4 (2.4)	1 (3.2)	1 (1.6)	2 (2.8)		
Operative time (min)	231.6 ± 73.9	241.8 ± 70.5	214.4 ± 59.8	242.2 ± 84.0	2.796	0.064
Estimated blood loss (ml)	61.1 ± 65.3	42.0 ± 13.1	52.9 ± 26.1	76.6 ± 93.7	3.990	0.020*^b^
Postoperative hospital days, median (IQR)	11 (9, 13)	11 (9, 13)	11 (10, 13)	10 (9, 13)	2.935	0.231
Histopathologic results						
Cholecystitis, n (%)	89 (53.6)	10 (32.3)	36 (57.1)	43 (59.7)	7.081	0.029*^b^
Cholangitis, n (%)	83 (50.0)	9 (29.0)	36 (57.1)	40 (55.6)	7.534	0.023*^ab^
Hepatic fibrosis, n (%)^#^	52/123 (42.3)	19/31 (61.3)	15/38 (39.5)	18/54 (33.3)	6.485	0.039*^b^

Abbreviation: IQR, interquartile range

* Significance level is < 0.05; ^a^*P* < 0.05 (group A vs. group B); ^b^*P* < 0.05 (group A vs. group C); ^c^*P* < 0.05 (group B vs. group C)

^**#**^ The item not checked in all patients. The data were presented as “number of measured patients.” Number in the parenthesis is percentage of measured patients

In terms of histopathological examination, group A was less likely to have cholecystitis than group C and less likely to have cholangitis than groups B and C (all *P* < 0.05; Table 2). Liver biopsy was performed in 31 patients (100%) in group A, 38 patients in group B (60.3%), and 54 patients in group C (75.0%), and the proportion of hepatic fibrosis was significantly higher in group A than in group C (61.3% vs. 33.3%, *P* < 0.05; Table 2).

### Postoperative complications

Early complications occurred in two patients (6.5%), six patients (9.5%), and 15 patients (20.8%) in groups A, B, and C, respectively. In group B, one patient had concurrent postoperative portal bleeding, pancreatic fistula, and pancreatitis and underwent revision surgery. Group C had a relatively higher complication rate, and the main early complication in this group was pancreatitis (n = 11, 15.3%). Late complications occurred in three patients (9.7%), six patients (9.5%), and 11 patients (15.3%) in groups A, B, and C, respectively. As a whole, cholangitis was the major late complication (n = 11, 6.6%). In addition, the incidence of various postoperative complications, both early and late, was similar among the three groups (all *P* > 0.05, Table 3).

**Table 3:** Postoperative complications of the three groups

	ALL (n = 166)	Group A (n = 31)	Group B (n = 63)	Group C (n = 72)	Statistical value	*P*-value
Early complication, n (%)	23 (13.9)	2 (6.5)	6 (9.5)	15 (20.8)	4.857	0.090
Bleeding	3 (1.8)	0 (0.0)	1 (1.6)	2 (2.8)	0.719	1.000
Bile leakage	4 (2.4)	0 (0.0)	2 (3.2)	2 (2.8)	0.703	1.000
Anastomotic stenosis	2 (1.2)	1 (3.2)	0 (0.0)	1 (1.4)	2.073	0.482
Pancreatic fistulae	2 (1.2)	0 (0.0)	1 (1.6)	1 (1.4)	0.654	1.000
Pancreatitis	16 (9.6)	1 (3.2)	4 (6.3)	11 (15.3)	4.274	0.131
Late complication, n (%)	20 (12.0)	3 (9.7)	6 (9.5)	11 (15.3)	1.132	0.548
Wound infection	2 (1.2)	0 (0.0)	1 (1.6)	1 (1.4)	0.654	1.000
Cholangitis	11 (6.6)	2 (6.5)	3 (4.8)	6 (8.3)	0.733	0.782
Pancreatitis	5 (3.0)	0 (0.0)	2 (3.2)	3 (4.2)	0.926	0.843
Pancreatic cyst	1 (0.6)	1 (3.2)	0 (0.0)	0 (0.0)	3.175	0.187
Adhesion ileus	1 (0.6)	0 (0.0)	0 (0.0)	1 (1.4)	1.489	1.000

### Dynamic changes of laboratory indicators

**Fig. 3:**
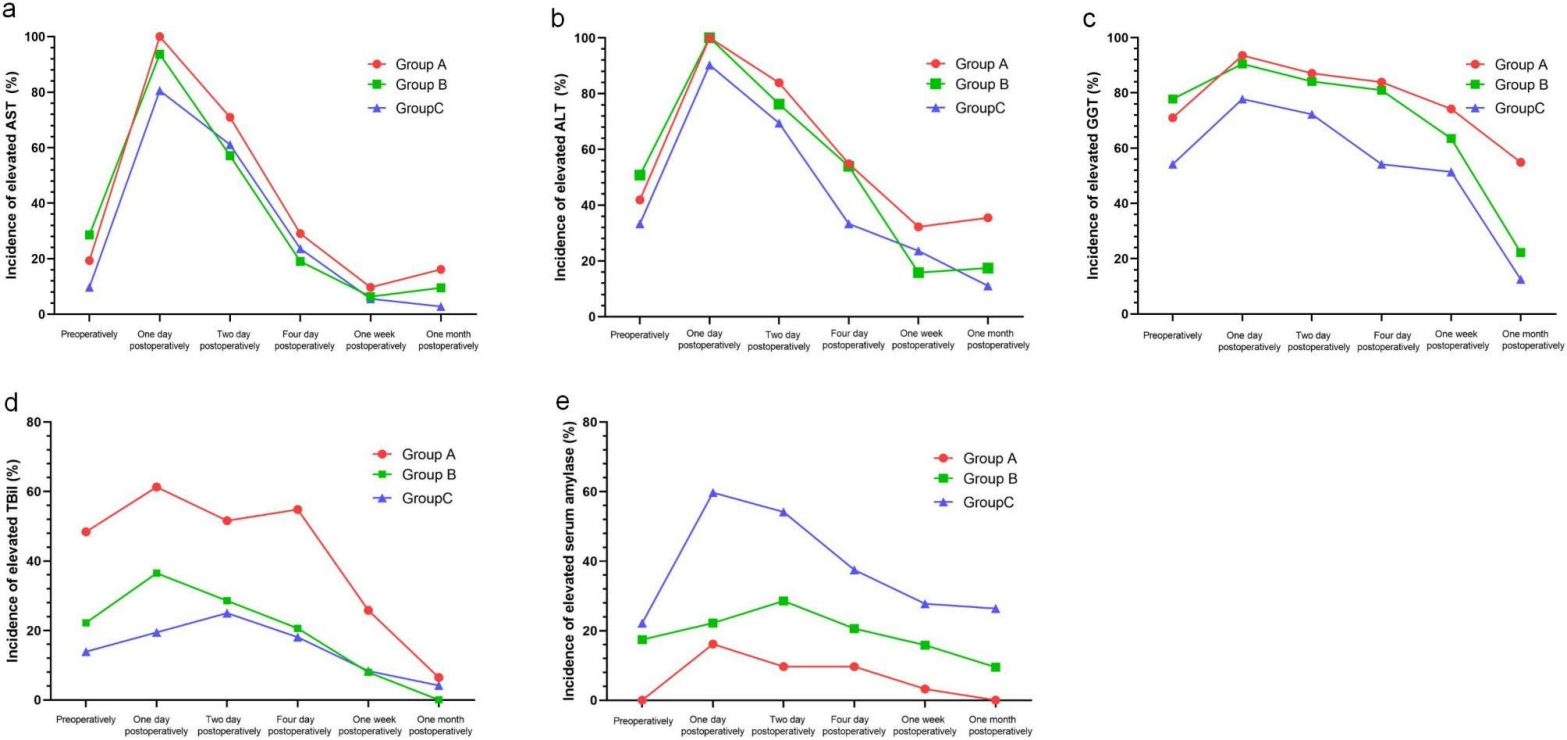
Dynamic changes of pre-, peri-, and post-operative laboratory indicators in the three groups. Abbreviations: AST, aspartate transaminase; ALT, alanine transaminase; GGT, gamma-glutamyl transpeptidase; TBil, total bilirubin

The three pediatric age groups showed differences in pre-, peri-, and post-operative laboratory indicators (Fig. 3). In general, for all three of the groups, the highest incidence of elevated indicators of liver function and amylase was observed on the first postoperative day. Whether preoperatively or postoperatively, group C was more likely to have elevated serum amylase, while groups A and B were more likely to present with abnormal liver function, including the increase in aspartate transaminase (AST), alanine transaminase (ALT), and gamma-glutamyl transpeptidase (GGT) levels. Except for the first postoperative day, the incidence of elevated GGT was greater than that of AST and ALT in the three groups. One month after operation, more than 50% of patients in group A still showed elevated GGT. During preoperative and perioperative periods, the proportion of patients with elevated total bilirubin (TBil) in group A was higher than that in groups B and C, but TBil recovered to a lower level in all of the groups at 1 month after operation (see Fig. 3).

## Discussion

PBM is mostly diagnosed in children and carries a risk of developing various pancreaticobiliary complications. In this study, we analyzed the pre- and post-operative characteristics of children of different age groups with PBM and found that these children presented differences in clinical manifestations, imaging measurements, pathological and surgical results, and laboratory indicators.

This study confirmed the predominance of females in PBM among all of the pediatric age groups, ranging from 4:1 to 3:1 [8, 9]. Infantile PBM patients were more likely to develop jaundice, while older children (≥ 1 year old) more commonly exhibited abdominal pain and vomiting. In addition, acute pancreatitis was mostly noticed in older children, especially those older than 3 years. In this study, no case of preoperative pancreatitis was seen in infantile patients with PBM. Obstructive cholangiopathy has been reported as the major cause of cholestatic jaundice in infants with PBM [15]. However, the main pathophysiological basis for the common symptoms in older PBM children was cholangitis caused by pancreatic juice flowing back to the bile duct or pancreatitis caused by hyperamylasemia [15, 16]. By considering these age-specific symptom patterns and underlying pathophysiological processes into account, clinicians can develop diagnostic and treatment strategies more effectively. For infants presenting with jaundice, attention should be paid to the possibility of obstructive cholangiopathy caused by PBM. For older children with PBM, increased vigilance towards symptoms of acute pancreatitis and cholangitis is warranted.

MRCP and CT are important imaging techniques that are helpful not only in the diagnosis of PBM but also in classifying PBM-related types and measuring structures adjacent to the pancreaticobiliary junction. In this study, older children with PBM (> 3 years old) showed a longer common channel than infantile patients. However, Itokawa et al. [17] explored the length of the common channel in 184 patients with PBM, aged 0–89 years, and found no relationship between age and the length of the common channel. This discrepancy could be explained by the different age spans of patients selected in the two studies. In any case, the length of the common channel is of concern because a relatively long common channel could be an important risk factor for the development of biliary tract cancer [18, 19]. Although the diameter of the CBD increases with age [12], infantile patients with PBM exhibited a larger maximum diameter of the CBD than older patients (> 3 years old). This result could explain why previous studies have found that abdominal mass was also the common symptom in infantile patients with PBM [9, 20, 21].

With the prolongation of disease duration, chronic cholestasis and bidirectional reflux can lead to the increased severity of biliary tract inflammation. Therefore, as our postoperative pathology results showed, older children with PBM were more likely to develop cholecystitis and cholangitis. Repeated and persistent inflammatory reaction could further cause severe and tight adhesion to the surrounding structures, which makes it difficult to dissect the bile duct wall and increases intraoperative bleeding. This could be the reason why older children, especially those aged over 3 years, were more likely to have greater intraoperative blood loss than infantile patients with PBM. These findings prompt consideration of whether additional preoperative treatments should be deployed for older children with PBM to minimize intraoperative risks. Specifically, whether anti-inflammatory therapies or other pharmacological interventions could be explored to address the chronic cholestasis and bidirectional reflux, aiming to mitigate biliary tract inflammation and severe adhesion and reduce the risk intraoperative bleeding. Although these strategies need to be further investigated and validated through prospective studies to establish the efficacy and safety, our findings could provide new ideas for a more personalized and effective management approach for pediatric patients with PBM. In addition, based on the Metavir scoring system, patients who underwent liver biopsy were diagnosed with or without hepatic fibrosis. Our study showed that hepatic fibrosis could occur in pediatric patients with PBM at all ages, but its incidence was higher in infants. Yang et al. [22] reported that age < 1 year at onset was an independent factor to predict hepatic fibrosis in children with PBM. Despite a higher incidence in infants with PBM, we observed that over 30% of older children also presented with liver fibrosis. As mentioned above, although the pathophysiological mechanisms differ at different ages, either obstructive cholangiopathy or reflux of pancreatic juice can contribute to the development of liver fibrosis [15]. Therefore, prompt surgical intervention should be performed, not only to prevent hepatic fibrosis but also to prevent biliary tract inflammation.

In this study, although there were no significant differences among the different age groups in terms of either early or late complications, the overall incidence was relatively higher in older children (> 3 years old). In that group, pancreatitis was the major early complication, and cholangitis was the main late complication. In addition, we analyzed the dynamic changes of pre-, peri-, and post-operative laboratory indicators, including serum levels of liver function indicators and amylase, in the three pediatric age groups. In general, whether preoperatively or postoperatively, younger patients were more likely to have abnormal liver function indicators, while older children were more likely to have abnormal serum amylase. Before and 1 month after operation, the incidence rates of elevated serum amylase in group C were around 20%, while there were no such cases in group A at both time points. This is consistent with the clinical symptoms and postoperative complication results described above. The development of digestive enzyme secretion is associated with age. The secretory response of the human pancreas to stimulation is acquired after the perinatal period, and exocrine pancreas continues to develop after birth. Pancreatic amylase expression is low at birth and rises slowly, reaching adult levels by the age of 2–3 years [23]. Therefore, in older patients with PBM, especially those over 3 years old, the level of serum amylase should be regularly monitored to timely detect and manage potential pancreatitis both before and after surgery. Given the susceptibility of infants with PBM to liver dysfunction, special attention should be paid to liver function markers, including AST, ALT, GGT, and TBil, during preoperative monitoring and postoperative follow-up. Previous studies have reported that serum GGT is an important biomarker in diagnosing biliary atresia and in predicting clinical outcomes [24–26]. In this study, except for the first postoperative day, the incidence of elevated GGT was greater than that of AST and ALT in all three of the groups. One month after operation, more than 50% of infants with PBM still showed elevated serum GGT. This finding indicated that serum GGT may be a more sensitive biomarker than AST and ALT to monitor liver function in children with PBM. However, further studies with long-term follow-up are needed to confirm this assumption.

This study has several limitations. First, this was a single-center study with a limited sample size. Second, this was a retrospective study with a relatively short follow-up. Third, this study did not assess the dynamic changes of laboratory indicators beyond 1 month after surgery due to differences in long-term follow-up time. Therefore, a multicenter prospective study with long-term follow-up is a necessary future endeavor.

## Conclusion

Due to different pathophysiological mechanisms, PBM manifests differently in children of different age groups. Infantile patients are more likely to present with jaundice, abnormal liver function indicators, and even liver fibrosis, which are mainly caused by obstructive cholangiopathy. However, in older children with PBM, long-term bidirectional reflux and hyperamylasemia are the main causes of cholangitis and pancreatitis, which result in abdominal pain and vomiting. Therefore, targeted management of PBM in children of different age groups should be carried out with paying due consideration to these differences.

## Data Availability

The datasets generated and/or analyzed during the current study are not publicly available due to ethical restrictions but are available from the corresponding author on reasonable request.

## Abbreviations

ALT: Alanine transaminase
AST: Aspartate transaminase
CBD: Common bile duct
CC: Choledochal cyst
CT: Computed tomography
GGT: Gamma-glutamyl transpeptidase
IOC: Intraoperative cholangiography
IQR: Interquartile range
MRCP: Magnetic resonance cholangiopancreatography
PBM: Pancreaticobiliary maljunction
SD: Standard deviation
TBil: Total bilirubin
